# Indicators of the dimensions of trust (and mistrust) in early primary care practice: a qualitative study

**DOI:** 10.1186/s12875-023-02098-2

**Published:** 2023-07-20

**Authors:** Allen F. Shaughnessy, Andrea Vicini, SJ, Mary Zgurzynski, Monica O’Reilly-Jacob, Ashley P. Duggan

**Affiliations:** 1 Department of Family Medicine, Tufts University School of Medicine, Boston, MA USA; 2 Theology Department, Boston College, 140 Commonwealth Ave, Chestnut Hill, MA USA; 3 William F. Connell School of Nursing, Boston College, 140 Commonwealth Ave, Chestnut Hill, MA USA; 4 Undergraduate Student, Boston College, 140 Commonwealth Ave, Chestnut Hill, MA 02467 USA; 5 Communication Department, Boston College, 140 Commonwealth Ave, Chestnut Hill, MA USA

**Keywords:** Trust, Qualitative, Primary care, Resident physicians, Family medicine, Reflective writing

## Abstract

**Background:**

Trust occurs when persons feel they can be vulnerable to others because of the sincerity, benevolence, truthfulness and sometimes the competence they perceive. This project examines the various types of trust expressed in written reflections of developing healthcare clinicians. Our goal is to understand the roles trust plays in residents’ self-examination and to offer insight from relationship science to inform the teaching and clinical work for better trust in healthcare.

**Methods:**

We analyzed 767 reflective writings of 33 residents submitted anonymously, to identify explicit or implicit indicators attention to trust or relationship development. Two authors independently coded the entries based on inductively identified dimensions. Three authors developed a final coding structure that was checked against the entries. These codes were sorted into final dimensions.

**Results:**

We identified 114 written reflections that contained one or more indicators of trust. These codes were compiled into five code categories: *Trust of self/trust as the basis for confidence in decision making*; *Trust of others in the medical community*; *Trust of the patient and its effect on clinician*; *Assessment of the trust of them exhibited by the patient*; and *Assessment of the effect of the patient’s trust on the patient’s behavior*.

**Discussion:**

Broadly, trust is both relationship-centered and institutionally situated. Trust is a process, built on reciprocity. There is tacit acknowledgement of the interplay among what the residents do is good for the patient, good for themselves, and good for the medical institution. An exclusive focus on moments in which trust is experienced or missed, as well as only on selected types of trust, misses this complexity.

**Conclusion:**

A greater awareness of how trust is present or absent could lead to a greater understanding and healthcare education for beneficial effects on clinicians’ performance, personal and professional satisfaction, and improved quality in patients’ interactions.

Where is the wisdom we have lost in knowledge?

Where is the knowledge we have lost in information?

-- From *The Rock*, T.S. Eliot

## Background

In healthcare, the yearning to be seen as an individual is particularly poignant because the relationship between clinician and patient is in many ways so intimate—and, at the same time, the learning and practice of relationships in clinical care can feel so distant. The vulnerability of sharing, with the resulting generation of wisdom, can get lost in biomedical measures, in knowledge, and information. In this project, we explore written reflections from developing healthcare clinicians to offer insight into the teaching and clinical practice of trust. Our project and data offer practical insight for teaching and improving trust in healthcare, including intrapersonal trust (trusting oneself) and interpersonal/relational trust (trusting colleagues and patients) and organizational trust (trusting healthcare systems and structures). We address implications of trust from relationship science and human communication research as well as practical implications of trust.

Across disciplines, trust is conceptualized as entailing some level of risk, uncertainty, or willingness to be vulnerable, and trust creates an expectancy about future behavior since a person must assume that a person, group, or organization will behave in a particular way [1]. Trust, at its core, is when a person feels they can be vulnerable to others because of the sincerity, benevolence, truthfulness and sometimes the competence they perceive of others [2]. People may extend trust to individuals, organizations, or societal structures that could act to further our interests or protect what we see as vulnerable. Trust often involves a transfer of power to a person or to a system [3]. Trust in organizations involves not only competence, but also openness and honesty, concern for stakeholders, reliability, and a sense of attachment or values that are aligned with our desires [4]. Healthcare professionals and patients know well when they experience trust in a healthcare interaction and system; they can rely on their healthcare team to be at their service and committed to promoting well-being [5].

Trust is usually bidirectional and relationship-based, built over time and across a series of interactions. Patients who experience high trust are less likely to second guess what healthcare professionals suggest as diagnostic follow-up, therapy, or lifestyle advice; an additional expert opinion might still be needed, but even that is an expression of a trust-based relationship [6]. Trust is usually vigilant and includes critical discernment, not implying dependence or surrendering independence [2]. Clinicians who feel they can trust and are trusted by patients feel recognized and appreciated for their clinical knowledge and competence and are confirmed in their ability to promote health, well-being, and flourishing in patients and within society [7]. Trust is a commodity that cannot be presupposed, but can be examined and promoted [8]. Trust also involves systemic and structural dimensions such that increasing trust in healthcare contexts can be beneficial in multiple ways—subjective and objective, relational and social, financial and organizational [9].Trust is a value in itself, and trust facilitates deeper relational interactions, continuity of care, quality of services provided, and facilitates opportunities for containing healthcare costs; on the other hand, when trust is lacking, dissatisfaction, disappointments, and frustrations appear to dominate and compromise healthcare experiences [2].

The effect of trust on the provision and receipt of healthcare is well documented. Better trust of physicians by patients lowers their perception of risk of treatment [10]. Patients with diabetes who trust their physician are more likely to follow suggested self-care guidelines [11]. Trust is also both a critical ingredient to and result of effective shared decision-making [12, 13]. Patients who do not trust their physicians are more inclined to make their own decisions rather than using a shared decision approach [14].

This article focuses on trust in an inductive way by examining various types and dimensions of trust identified in reflective writings from developing healthcare clinicians attending to their experiences interacting with patients, colleagues, and staff. Written reflections offer insights into their understanding of trust and how trust impacted them. Our goal is to understand the various roles trust play in residents’ self-examination and to offer implications for teaching and improving trust in healthcare.

## Methods

This project began as an effort to introduce reflective writing and to improve reflective writing skills of family medicine residents in a single residency in the United States. The 33 residents were given regularly scheduled time to write these reflections into an internet-based database. They were not required to write the reflection at this time but could access the database at any time. They were not given a prompt but were asked to give each entry a title and link to clinical topic where appropriate. The reflections were not graded or evaluated in any way and were available only to the resident. Most (94%) of the residents participating in this project were women and were at all levels of residency education. This percentage is consistent with 94% of the Family Medicine residents being women. A total of 767 reflections were written over 18 months and were collected for this project.

### IRB approval and ethical protections for participants

We obtained Institutional Review Board approval from two institutions where the researchers are affiliated. First, the project was approved as exempt and with waiver of written informed consent by the Cambridge Health Alliance Institutional Review Board (first author’s affiliation) and then by Boston College (other authors’ affiliation) under the classification of education research. All methods were carried out in accordance with regulations that apply to exempted research. Reflection entries were de-identified by a researcher not involved with participants (AD) before the analysis, other than being categorized by year of training. This analysis was performed after all participants graduated from the training program. In addition, examples from the reflections in this paper are aggregated or paraphrased in a way to disguise identification with a specific residency or medical resident.

### Analysis

Analysis of the reflections began with a research assistant identifying explicit uses of the word “trust” in the writings. One author read through all reflective entries. Using an inductive method to develop a framework of the underlying structure of experiences in the data [15], this author identified entries with explicit or implicit indicators of reporting or reflecting on issues of trust. These entries served as the basis for analysis. A second author read through all these entries to further identify meaning units. Both authors developed separate coding structures. After discussion with a third author, the final coding structure was developed and then checked by applying it to the entries.

Hence, an interdisciplinary research team read through the entire data set and identified, through a-priori consensus process, trust as a content area to be qualitatively examined. Within reflections addressing trust, themes were systematically and inductively derived from the data. Research team members read through the entire data set each independently and then as a group, identifying themes as well as latent and manifest content areas that emerged from the reflections [16]. Emerging themes and occurrences of communication were quantified/counted with the reflective entry as the unit of analysis and then sorted qualitatively and independently by one of the research assistants grouping together under higher order headings.

Similar to grounded theory as a philosophical approach and research method, the qualitative content analysis allowed explanatory framework to be developed through systematic gathering and analysis of data such that explanations are grounded in the data [17]. The method is in contradistinction to the hypothetico-deductive model usually used in science, in which a predetermined theory or hypothesis is developed and then tested using any of a variety of study designs. We used an iterative process of systematically and inductively identifying themes and creating categories within the reflections [16]. An undergraduate research assistant from Boston College served as the initial primary coder (MZ); she read and categorized all of the occurrences of trust independently and processed identified themes on a weekly basis with the other authors. In cases of disagreement, or questions, the team shifted and reorganized until consensus was reached. As an additional guard against arbitrary decision-making, we each re-analyzed the data together after developing categories to minimize force-fitting data.

### Interpretation

All authors have extensive experience with qualitative research and implications of social construction of interpretations in research [18]. One researcher has a background in medical education, another has a background in human communication research, and the third researcher has a background in medicine and bioethics. Each investigator thus applied a unique frame of reference in the analysis. We used a reflexive approach to examine our own beliefs and judgments [19]. At each point in the process–after entry selection, initial coding, secondary coding, and final coding structure checking–the research group independently collected notes on their thoughts and then met to discuss and reflect on the findings to develop a framework based on consensus for analysis.

## Results

We identified 114 written reflections that contained one or more indicators that the writer was considering trust, either implicitly or explicitly. These codes were compiled into five dimensions.

*Trust of self/trust as basis for confidence in decision making* (n = 50 entries). Trust of self is demonstrated by an explicit assessment of one’s own knowledge, skill, or competence, which leads to a determined assessment of one’s competence to perform clinically. These entries reflected their explicit examination of their own knowledge, skill, or competence, or their assessment of their confidence, using phrases such as, “I don’t feel like I did a fantastic job,” “I don’t have much experience with…,” “I am quickly being reminded of the overwhelming amount of medical information that I do not know,” and, “it was a classic case but I hesitated making this diagnosis initially”. One resident wrote, “[I] just saw a challenging [patient]; amazing how powerful the creepy vibes are. [In] these scenarios I have to stick to my instincts because they are probably right.” In addition, they used entries to capture notes from lectures or direct lifts from information resources may indicate their acknowledgement of the perceived tenuousness of their knowledge.

*Trust of others in the medical community* (n = 24 entries). Trust in the community involves the ability to rely on others to aid in one’s care of a patient. These entries commented on residents’ level of trust in the healthcare systems or their colleagues to support the care they wished to provide for their patients. Some entries reflected an assessment of specialists or supervisors. For example, one person wrote, “well, I don’t feel as inadequate about my adolescent interview techniques after observing the ‘professionals’ suffer through a non-cooperative teenage patient.” Another wrote, “She saw [a specialist] who recommended using urea cream… I am a little skeptical of this.” Some entries reflected on the healthcare system in general: “Some of my biggest concerns over my limited time in residency stem from the inefficiency [and occasional un-safety (sic.)] of the systems we have in place”; “Peds was a circus too. The floor is so inefficient it should be featured in some business PowerPoint entitled ‘how not to do things.’ Some entries also questioned the value they perceived they would receive from specialists if they were to refer patients to them: “Thought about urology but didn’t think it would help much.”

*Trust of the patient and its effect on clinician* (n = 9 entries). These entries examined residents’ trust of a particular patient and how it affected them, either personally or their decision making. One resident reflected on a patient visit: “I felt torn, and a little defeated. Defeated because I knew that I would give this woman [an opioid] … Defeated because I didn’t want to do that, I wanted to talk to her longer, understand the history, understand more what her stressors were, and discourage the dependence… Torn because I feel for her, and that she has to manipulate and lie in order to feed her addiction. Torn because her provider, me, does not trust her, and that’s not a nice thing to feel.”

Particularly poignant entries focused on patients requesting medication for pain, (regarding a patient asking for a specific scan): “I guess I’m left with a feeling where it is hard for me to know how to act or think when I have patients who I’m not sure are telling me the truth. I want to believe them and help them and get to know them, but I’m afraid that they’re just using me.”

*Assessment of the trust of them exhibited by the patient* (n = 19 entries). Developing clinicians seemed to implicitly grasp the role of relationship development in their care, often commenting on their assessment of the trust or lack of trust exhibited by a particular patient. Several reflective entries seemed to marvel at the institutional trust conferred on clinicians, of patients not questioning them as a doctor or as their healthcare clinician. As an example, “Patients fully respected the title and had all the expectations of me that they would have of my preceptors. This is both exciting and scary.”

Developing clinicians also reflected on patients who did not seem to trust them: “I can’t believe she accused me of being racist of all things!”; and, “Right there in one fell swoop, I felt that I had cut ties with the mother and her daughter.”

*Assessment of the effect of the patient’s trust on the patient’s behavior* (n = 12 entries). Residents also commented on their assessment of the connection between the patient’s behavior and the resident’s assessment of the patient’s trust in them: “I do think I established a good rapport with the patient, and that she felt comfortable talking to me about her depression.” Commenting on a patient’s compliment, one resident wrote, “I’ve spent some time talking with him about his leg pain (which he is imminently worried about and is putting highest on the priority list, above his heart rate of 20, ARF, and serious need for a pacemaker) … Unlike all his other doctors, apparently, I could pretend to care, and take a little time to write some orders for pain meds and capsaicin cream. And, apparently, that made all the difference.”

## Discussion

Based on inductive analysis to develop an explanatory framework for the underlying structure of experiences [15] this article develops an explanatory framework for dimensions of trust (and mistrust) in early primary care practice based on healthcare clinicians’ reflections written over an academic year. This article identifies direct indications of trust, as well as.

more subtle cues that suggest concern or mistrust. Analysis provides evidence for five dimensions of trust including trust of self/trust as basis for confidence in decision making, trust of others in the medical community, trust of the patient and its effect on clinician, assessment of the trust of them exhibited by the patient, and assessment of the effect of the patient’s trust on the patient’s behavior. Examples indicate the importance of understanding trust through implicit or subtle cues in addition to explicit statements. Implicit cues indicative of trust (or mistrust) are important, because when we get to a point where we say “you can trust me,” or when we ask whether we can trust someone, we have missed something fundamental in the relationship or institutional context. Identified dimensions of trust extend previous research where measures of trust are based on biomedical competence and attentiveness indicative of health service quality [20].

Each concrete illustration from healthcare clinicians’ written reflections situate and exemplify the dimensions of trust that the authors identify. In healthcare interactions, diverse and even contrasting ways of experiencing trust (or mistrust) can occur at the same time or and can be spread in daily practice. In other words, trust is built on reciprocity over time, and, as with every relational process, trust is dynamic, fluid, and changing. Hence, trust can evolve; trust can be challenged and threatened. Multiple dimensions of trust facilitate the relationship between patients and healthcare professionals and vice versa; mistrust, on the contrary, damages the relationship.

The written reflections provide evidence that trust belongs to the realms of personal experience and of relationality. Hence, trust engages one’s identity. In particular, both patients and healthcare professionals can grow in trusting one another–for example, by sharing something about what matters in their lives–and this sharing reinforces their personal and professional identity. Conversely, when trust is abused, a person loses the ability to trust other people or institutions. Reflecting on trust requires a person to consider the experiences that influenced and shaped trust, how that person can recognize and name those experiences, how the person is still suffering from those experiences, how the person can still be trapped in mistrust, how the person has healed from mistrust, how previous experiences with trust (or mistrust) have empowered or disempowered the person.

### Dimensions of trust

Examples in this article provide evidence for broader dimensionality of trust in healthcare than in previous research. Trust can begin as unidirectional and anonymous, like when we strap ourselves into a seat of a jet that will hurtle us from one place to another at 900 km per hour. We place our trust in a pilot, a piece of machinery, and the law of physics, perhaps initially without a second thought. Trust can be *intra*personal–I trust or do not trust myself–or *inter*personal–I can trust or not trust and, conversely, they can trust or not trust me. I can gain or lose the trust of another and gain or lose trust in the other.

Agents of institutions can be trusted, or mistrusted, based on institutional structures, organizational arrangements, dynamics in interactions with people, employees, groups, and communities. Trust, or mistrust, in science (medicine, drugs, public health measures, diagnostic tools, therapies), may be differ between clinicians and patients. Corruption that draws a social institution away from its mission and undermines its integrity can lead to broad mistrust of both the institution and its members [21]. We see this played out in the mistrust of agents of government, information sources, and other institutions. The focus on institutional trust in this article extends the framework of previous research describing mutual trust in terms of continuity of care or familiarity with the doctor [22].

Examples indicate how trust depends on truth but, more importantly, the trustors’ *perception* of the degree of truth. Trust can be contextual, e.g., I might trust my family doctor to understand my emotions but not to remove my appendix. Trust may ebb and flow, reinforced or negated by relationships. Mistrust can come from communication difficulties [22]. Trust can be earned (or not) or sometimes is granted provisionally without demonstration, especially when the need for trust is high. Patients may trust their healthcare clinician whom they have just met because they trust the profession. This lease on trust, based on the trust of the institution, will be ratcheted up or down based on additional experiences.

Healthcare clinicians trust conclusions derived from their education or research findings (evidence-based medicine) until their knowledge is called into question by their subsequent experiences. In addition, they need to, based on relational clues, trust both in the patient and in their own ability to address explicitly stated concerns as well as hints or cues of what the patient wants (and does not want).

### Trust and relationships in healthcare

The process of dyadic interdependence in navigating trust is a communication process—sometimes that process translates into reciprocity in the interaction and in building and maintaining a relationship, even in power imbalanced or role-based positions. I am trusted, I trust. If I am not trusted, I may suspend my ability of trusting until trust can be restored, or I lose my ability of trusting until I heal from experiences of mistrust. As part of the diagnostic process and therapeutic alignment, healthcare clinicians need judge what the patient says and demands, what the patient needs, what the patient does not want, what the patient is unable to see. Experiences can reinforce trust in oneself, others, and institutions.

Trust is not only given: “I trust someone or something.” Trust is also received: “I am trusted by someone, for who I am, for what I am doing, for something I did, and for what I did not do.” In their reflective entries physicians indicate how the trust they received from the medical guild during their training, and from colleagues, staff, and patients in their daily practice, made them trust themselves more. In a way, trust builds trust: the way others trust us–because we are trustworthy–builds our ways of trusting ourselves as human beings and as professionals. Of course, trust is also nourished by formation, training, and experience.

Physicians describe how further study, repeated practice, attentive reflection, and critical analysis strengthen their trust in what they are able of doing, in their skills, and in their ability of learning from their omissions, uncertainties, reductive and limited diagnostic differential possibilities, and mistakes. Hence, trust is transformative, transforming in positive ways one’s being and doing. On the contrary, if one’s trust has been abused, the ability to trust other people or institutions might be lost. Such a loss can be provisional and temporary, waiting to see whether it could be possible to restore trust and what that would entail.

### Trust in the institution of medicine

Trusting relationships are influenced by context. There is a reciprocity between trust between clinician and patient and how the institutions–medicine, science–are trusted. There is tacit acknowledgement of the interplay among what the residents do is good for the patient, good for themselves, and good for the medical institution. There is also, in the reflections, a marveling, especially by residents just beginning their training, of an implicit trust in their competence and benevolence because they, though previously not known to their patients, are part of the institution of medicine. They acknowledge that the trust given to them is only leased–that patients will provisionally trust them until such time that the residents’ behavior will confirm and reinforce this trust, or disprove it.

Institutions influence the healthcare practice and the agency both of healthcare professionals and of patients. Institutions can contribute to creating a trusting environment that fosters trust or they can jeopardize the development of trust (e.g., because of bureaucracy, because of costs, because of limited access, because of racial dynamics, because of hindrances to communication when patients’ languages and worldviews are not understood). Given the frequent and off-hand way of commenting on it, residents seemed to see trust development of them by the patient as being central to their effectiveness, building on the institutional trust that served as the basis for the visit.

Residents also exhibited or directly commented on their trust in themselves, either regarding their psychological suitability to provide care (either at that moment or in general) or in their clinical knowledge and prowess. For the latter, this conscious incompetence with the resulting lack of confidence is developmentally appropriate and a rationale for prolonged supervised practice. Trust in an institution derives from current and past experiences with that institution, what it does and did to me and to others, and from what does not do and did not do to me and to others; from its structures, organizational arrangements, dynamics in interactions with peoples, employees, groups, and those living in the surrounding territory.

Moreover, members of minorities know too well that trust goes beyond the personal relationship here and now. One could say that the “weight of history”–particularly in the case of histories of racial discrimination, abuses, and violence–burdens the possibility of trust, of trusting another, a healthcare professional, a healthcare institution, even the diagnostic and therapeutic processes. To heal trust and to restore trustworthy relationships demands more than good intentions, a strong will, and personal efforts. Social and institutional reparative and restorative processes leading to acknowledging those systemic and structural violations of trust are needed to create the conditions for social justice that are required to earn and experience trust.

The weight of personal histories matters too; for example, when one considers what concerns substance abuse. Because of these personal histories trust is challenged, in diverse ways, both by the healthcare professional and by the patient: the patient might not be trusted in her requests for more drugs, and the physician might not trust that giving those drugs will be beneficial for the patient. As personal accounts indicate, trust is elusive and maybe cannot be experienced in constrained and inhibiting relational dynamics.

### Trust and communication behavior

Whether one focuses on intrapersonal understanding, interpersonal relationships, or building trust in organizations, trust is built over time. In the relationship science literature, a spiral theory of trust purports that, first, “trust, once established, remains relatively fixed, but also spirals over time to increase or decrease trust in response to verbal and nonverbal behavior of participants” [1]. This functional approach allows one to specify key communication goals and desired outcomes (e.g., building trust, shared decision-making to include patients’ values, or managing uncertainty) that need to be accomplished for quality healthcare. Second, this functional approach embraces the notion that communication to build trust is essentially goal-oriented and aims at achieving outcomes through communication that contribute to improving, or sustaining, patients’ health and well-being. Finally, such theoretical grounding helps predict how to reach identified goals.

### Limitations and future directions

Although the identified dimensions of trust provide evidence for a broader conceptual foundation than in previous healthcare research, we recognize limitations in our study. First, written reflections came primarily from women working in early careers in primary healthcare and may be limited in the extent to which they represent men’s views or the views of non-binary identifying healthcare clinicians. Second, data come from an urban healthcare clinic in a large Northeastern city in the United States and therefore cannot claim to represent views of other cultures. For example, previous research indicates that trust in physicians in China appears quite low, and that Chinese patients with higher education and medical insurance indicate higher trust in physicians [23] Future research should elucidate dimensions of trust including nonverbal communication behavior indicative of trust (and mistrust).

## Conclusion

Like many, if not most, medical education curricula and programs, the role of trust and the means of cultivating and assessing it are not part of the formal teaching in the residency program from which this sample of residents was drawn. Yet, trust seems to be acknowledged informally in healthcare education as witnessed by the explicit mentions of trust by these residents. Allowing emerging physicians to develop insight into the role of trust in their practice may be an unintended result of this reflective writing requirement. Perhaps the formal exploration of the role of trust in healthcare is an appropriate educational goal for healthcare curricula.

## Data Availability

The datasets generated and/or analyzed during the current study are not publicly available due to the fact that the reflective entries come from an educational module and not technically a data set, but are available from the corresponding author on reasonable request.
